# A City Too Busy to Remember? Aging, Structural Violence, and the Politics of Forgetting in Atlanta’s Gentrification

**DOI:** 10.1093/geroni/igaa057.1266

**Published:** 2020-12-16

**Authors:** John Pothen, Keland Yip, Ellen Idler

**Affiliations:** Emory University, Atlanta, Georgia, United States

## Abstract

Can forgotten stories from the past inform a city’s future? As older adults continue to live longer and comprise more of the population than ever before, the suitability of gentrifying spaces for older adults aging in place is increasingly important. Critical theories of gentrification argue that remembering the experiences of older adults in this context - experiences of suffering, resilience, and structural violence - is essential to promote changes in support aging in place. In this study, we tell a story of individual experiences, structural violence, and aging in the ongoing gentrification of one neighborhood in southwest Atlanta. We construct this narrative through a qualitative analysis of 1,500 local newspaper articles from 1950 to the present day and 10 in-depth interviews with ex-residents of the neighborhood aged 65-87. Drawing on the theory of planetary rent gaps, we frame gentrification as a class struggle between property-owners and working class residents. We highlight the city government’s role as a facilitator for property-owners through projects including the Model City initiative, preparation for the 1996 Olympics, and ongoing development surrounding the Atlanta BeltLine. We show how these projects have affected the prospects for aging in place in general and, specifically, by affecting access to healthcare services. We share this story in an effort to combat the politics of forgetting and to inform a richer, more inclusive, and more equitable future for gentrifying spaces.

